# Prediction of mortality risk in patients with peripheral artery disease using interpretable machine learning

**DOI:** 10.3389/fcvm.2026.1879240

**Published:** 2026-08-10

**Authors:** Yifei Li, Qiang Zhang, Wenxin Zhao, Qingyuan Sun, Yue Wu, Jiayi Song, Jianan Zhang, WenQi Liang, Zuoguan Chen, Lishuang Guo, Sheng Yan

**Affiliations:** 1 Department of Vascular Surgery, The Second Hospital of Shanxi Medical University, Taiyuan, China; 2 Department of Vascular Surgery, The Ninth Clinical Medical College of Shanxi Medical University (Taiyuan Central Hospital), Taiyuan, China; 3 Department of Vascular Surgery, Beijing Hospital, National Center of Gerontology, Institute of Geriatric Medicine, Chinese Academy of Medical Sciences & Peking Union Medical College, Beijing, China; 4 Graduate School of Chinese Academy of Medical Sciences & Peking Union Medical College, Beijing, China

**Keywords:** clinical outcome, machine learning, neutrophil-to-lymphocyte ratio, peripheral artery disease, sample size

## Abstract

**Background:**

Patients with peripheral artery disease (PAD) face high postoperative mortality risks, necessitating precise risk stratification. While machine learning offers superior performance, its black-box nature limits clinical utility, and the prognostic value of the neutrophil-to-lymphocyte ratio (NLR) remains controversial.

**Methods:**

A total of 610 surgically managed PAD patients were enrolled (median follow-up: 4 years) and randomly split into training (70%) and test (30%) sets. Six machine learning algorithms were constructed and optimized. Model performance was evaluated using area under the receiver operating characteristic curve (AUC) and decision curve analysis (DCA). The sHapley additive exPlanations (SHAP) were employed for model interpretation and visualizing nonlinear relationships.

**Results:**

The random forest model achieved optimal performance (test set AUC = 0.814) with significant clinical net benefit. SHAP analysis identified age, prothrombin activity, and Rutherford classification as top predictors. Notably, while multivariate Cox regression failed to identify NLR as a linear predictor, SHAP dependence plots revealed a distinct nonlinear pattern: risk contribution increased sharply at low standardized NLR values before plateauing.

**Conclusion:**

We established an interpretable random forest model for predicting postoperative mortality in PAD. By integrating SHAP analysis, this study validates the nonlinear prognostic significance of NLR and demonstrates how explainable ML can complement traditional statistics for individualized risk assessment.

## 1 Introduction

Peripheral artery disease (PAD) has become the third most prevalent atherosclerotic disease following coronary heart disease and stroke, affecting an estimated 230 million adults globally (1). PAD is associated with elevated mortality, with 5-year mortality rates ranging from 10% to 20% (2). In China, the burden of PAD is escalating due to an aging population and increasing prevalence of metabolic risk factors. PAD is associated with significant morbidity and mortality, with postoperative mortality rates remaining high despite advances in endovascular techniques. Despite advances in endovascular technologies and expanded therapeutic strategies, postoperative mortality remains high, highlighting the critical need for accurate mortality prediction to guide individualized treatment decisions (3, 4).

From a pathophysiological standpoint, PAD is an atherosclerotic disease with multifactorial origins (5). Among these, inflammatory processes play a central role in disease progression in mediating its progression (6). The inflammation triggers the release of proinflammatory cytokines and an increase in neutrophil counts (7). Accordingly, the neutrophil-to-lymphocyte ratio (NLR) has emerged as a reliable marker for evaluating systemic inflammation across cardiovascular diseases, malignancies, and chronic obstructive pulmonary disease (2, 5–8). Mounting evidence also links elevated NLR to adverse clinical outcomes in patients with PAD (9).

Nevertheless, the independent prognostic value of NLR for long-term outcomes in PAD remains controversial (10, 11). Moreover, most existing studies rely on traditional statistical approaches (12). Conventional risk-stratification tools including Cox regression and logistic regression have shown limited performance in PAD, largely because they cannot adequately capture complex nonlinear relationships (13).This limitation is particularly evident in modeling inflammatory responses, which inherently exhibit threshold effects that linear models systematically miss (14). Furthermore, many existing studies suffer from small sample sizes or heterogeneous cohorts, limiting the generalizability of their conclusions, especially to domestic clinical settings where patient characteristics and comorbidities may differ.

Machine learning (ML) models excel at handling high-dimensional and nonlinear data, and have demonstrated superior predictive performance in cardiovascular risk stratification (15, 16). Yet the black-box nature of ML impedes clinical interpretability and clinician trust, slowing real-world translation (17). In recent years, explainable artificial intelligence (XAI) has emerged as a solution to this challenge (18). The sHapley additive exPlanations (SHAP) framework uses game theory to quantify the contribution of each feature to individual predictions, rendering ML decision-making transparent and clinically actionable (16). Despite these advances, few studies in PAD mortality prediction have systematically integrated multi-algorithm comparison, decision curve analysis (DCA), and in-depth XAI (19).

To address these gaps, this study aimed to construct and validate an interpretable ML model for predicting postoperative all-cause mortality in PAD patients. Leveraging a substantial single-center cohort from a tertiary care hospital in China (*n* = 610), we systematically compared six ML algorithms. The primary objectives were to: (1) establish a high-performance predictive model tailored to the domestic PAD population; (2) utilize SHAP analysis to resolve the controversy surrounding the prognostic value of NLR by elucidating its nonlinear relationship with mortality; and (3) demonstrate how interpretable ML can complement traditional statistics in clinical research.

## 2 Methods

### 2.1 Study population and data collection

This retrospective cohort study included 748 patients diagnosed with PAD (ABI ≤0.9 and confirmed by CT) who received surgical intervention at the Second Hospital of Shanxi Medical University from January 1, 2018, to December 31, 2025. Of the initially enrolled 748 patients, 138 were excluded due to loss to follow-up, with a loss to follow-up rate of 18.4%. A total of 610 eligible patients were finally included, and all participants completed the follow-up without additional dropout during the observation period. Inclusion Criteria: 1) Age ≥18 years; 2) PAD clinically and imaging-confirmed; 3) Receiving open surgery or endovascular intervention; hybrid surgery was also included, and cases with unrecorded surgical types were retained for analysis; 4) Completed comprehensive preoperative laboratory tests. Exclusion Criteria: 1) Patients with acute limb ischemia were strictly excluded, and all enrolled cases presented with chronic peripheral artery disease; 2) Severe infection or inflammatory disease within 4 weeks preoperatively; General infection was defined based on typical clinical manifestations, elevated white blood cell count, procalcitonin and C-reactive protein combined with etiological evidence. Severe infection was diagnosed in accordance with the 2021 Sepsis-3 criteria, defined as infection complicated with organ dysfunction (SOFA score ≥2) or progression to septic shock. Specifically, severe infections included severe pneumonia, necrotizing soft-tissue infection, sepsis, urinary tract infection with systemic inflammatory response, and other systemic infections requiring intravenous antibiotic therapy or inpatient anti-infective treatment within four weeks prior to surgery. Patients with localized mild infection without systemic inflammatory response or organ impairment (e.g., superficial mild skin infection) were not excluded; 3) Comorbid hematologic disease or malignancy; 4) Comorbid other severe organ failure; 5) Ongoing use of drugs affecting blood cells (e.g., glucocorticoids, immunosuppressants); 6) Loss to follow-up. Ultimately, 610 patients were included in the study, with the primary endpoint being all-cause mortality.

The research team established an electronic medical record database to systematically collect preoperative baseline characteristics of the patients. The collected data included: 1) *demographic characteristics*: age, gender, and body mass index (BMI). 2) *Clinical features:* Rutherford classification and Fontaine stage. Intervention indications were classified as intermittent claudication and chronic limb-threatening ischemia (CLTI), with 219 (35.9%) and 391 (64.1%) patients respectively. 3) *Comorbidities*: smoking history, diabetes, hypertension, coronary artery disease, and chronic kidney disease staging. 4) *Cardiac function*: left ventricular ejection fraction (LVEF%). 5) *Laboratory parameters*: Complete blood count: white blood cell count (WBC), neutrophil count (NEUT), lymphocyte count (LYMPF), platelet count (PLT), hemoglobin (HGB), and red cell distribution width (RDW). Biochemical indicators: total protein (TP), creatinine (Cr), urea (Urea), triglycerides (TG), and total cholesterol (TC). Coagulation function: D-dimer (D_D), fibrinogen (FIB), prothrombin time activity (PTA), and activated partial thromboplastin time (APTT). Glucose-related indicators: fasting blood glucose (FBG) and glycated hemoglobin (HbA1c). 6) *Surgery-related characteristics*: American society of anesthesiologists (ASA) classification. Surgical modalities were categorized into four predefined groups: endovascular intervention (323 cases, 52.95%), open surgery (204 cases, 33.44%), hybrid surgery (61 cases, 10.00%), and unknown surgical type (22 cases, 3.61%). The chi-square test revealed no significant difference in the distribution of surgical types between survivors and non-survivors (*P* = 0.292). According to balloon dilation and stent implantation sites, arterial lesions were categorized into aortoiliac lesions (common iliac, external iliac, internal iliac arteries), femoropopliteal lesions (common femoral, deep femoral, superficial femoral, popliteal arteries), below-the-knee lesions (tibioperoneal trunk, anterior tibial, posterior tibial, peroneal, foot arch arteries), and other arterial lesions. Among patients with clear lesion records, 480 (77.3%) had femoropopliteal lesions, 259 (41.7%) had below-the-knee lesions, 202 (32.5%) had aortoiliac lesions, and 7 (1.1%) had other lesions. A total of 617 balloon dilation procedures were performed; the most frequent sites were superficial femoral artery (398 times, 64.5%), popliteal artery (219 times, 35.5%), anterior tibial artery (175 times, 28.4%), peroneal artery (133 times, 21.6%), posterior tibial artery (129 times, 20.9%), common iliac artery (100 times, 16.2%), and external iliac artery (75 times, 12.2%). A total of 543 stent implantation procedures were conducted, including femoropopliteal artery (324 times, 59.7%), aortoiliac artery (205 times, 37.8%), below-the-knee artery (10 times, 1.8%), and other sites (4 times, 0.7%). Notably, all enrolled patients received standardized guideline-conformant perioperative medication regimens including statins, antiplatelet agents, and anticoagulants after admission in our center with unified clinical protocols. Given the consistent and routine medication strategy for the entire cohort, detailed individualized medication dosages, dynamic adjustment strategies, and long-term medication adherence data were not retrospectively recorded. A complete list of all variables is provided in Table 1.

**Table 1:** Baseline characteristics of participants.

Characteristics	Alive	Death	*P*. value
n	547	63	
Age (mean (SD))	67.38 (10.09)	74.49 (8.72)	<0.001
BMI (median [IQR])	23.03 [21.16, 24.82]	22.96 [20.91, 24.56]	0.709
LVEF% (median [IQR])	65.00 [62.95, 67.00]	64.00 [63.00, 66.00]	0.139
ABI-right (median [IQR])	0.64 [0.51, 0.79]	0.63 [0.47, 0.74]	0.849
ABI-left (median [IQR])	0.60 [0.47, 0.76]	0.56 [0.43, 0.65]	0.062
WBC (median [IQR])	6.97 [5.75, 8.51]	7.92 [6.41, 10.29]	0.005
RBC (median [IQR])	4.45 [4.08, 4.87]	4.14 [3.63, 4.68]	0.006
HGB (median [IQR])	138.00 [125.00, 152.00]	130.00 [113.50, 142.50]	0.001
HCT %(median [IQR])	41.2 [37.5, 45.6]	41.4 [37.8, 45.8]	0.087
MCV (median [IQR])	93.40 [90.20, 96.65]	90.90 [88.10, 95.70]	0.026
MCH (median [IQR])	30.90 [29.70, 32.10]	29.80 [28.70, 31.35]	0.003
MCHC (median [IQR])	330.00 [323.50, 336.00]	327.00 [320.00, 332.00]	0.014
RDW-CV (median [IQR])	13.00 [12.50, 13.70]	13.50 [12.96, 14.30]	0.001
RDW-SD (median [IQR])	44.50 [42.30, 47.20]	45.70 [42.35, 49.40]	0.139
PLT (median [IQR])	220.00 [175.50, 269.00]	238.72 [176.50, 298.50]	0.264
MPV (median [IQR])	10.20 [9.50, 11.00]	10.00 [9.50, 10.80]	0.724
PDW (median [IQR])	13.70 [11.40, 16.00]	13.00 [10.80, 16.00]	0.490
ESO (median [IQR])	0.14 [0.08, 0.24]	0.13 [0.08, 0.26]	0.797
BASO (median [IQR])	0.03 [0.02, 0.05]	0.03 [0.02, 0.05]	0.982
NEUT (median [IQR])	4.40 [3.37, 5.64]	5.40 [3.97, 7.37]	<0.001
LYMPH (median [IQR])	1.78 [1.35, 2.28]	1.47 [1.10, 2.03]	0.002
NLR (median [IQR])	2.40 [1.74, 3.44]	3.60 [2.68, 5.37]	<0.001
MONO (median [IQR])	0.49 [0.39, 0.61]	0.53 [0.42, 0.64]	0.189
TP (median [IQR])	65.50 [62.00, 69.50]	64.60 [60.50, 69.05]	0.296
ALB (median [IQR])	38.70 [36.25, 41.00]	36.00 [30.80, 39.15]	<0.001
GLB (median [IQR])	26.90 [24.10, 29.80]	30.30 [27.10, 34.02]	<0.001
ALT (median [IQR])	17.80 [12.70, 25.15]	18.90 [12.05, 26.20]	0.901
AST (median [IQR])	20.40 [16.75, 25.00]	19.80 [15.75, 28.19]	0.651
Cr (median [IQR])	71.00 [61.00, 82.95]	78.55 [64.00, 102.50]	0.005
FBG (median [IQR])	6.39 [5.08, 7.96]	6.57 [5.00, 7.83]	0.839
Urea (median [IQR])	6.00 [4.90, 7.40]	6.70 [5.40, 7.80]	0.048
Ca (median [IQR])	2.25 [2.17, 2.33]	2.18 [2.08, 2.30]	0.002
TC (median [IQR])	4.19 [3.71, 4.86]	3.94 [3.48, 4.62]	0.026
TG (median [IQR])	1.57 [1.21, 1.96]	1.47 [1.19, 1.77]	0.079
LDL-C (median [IQR])	2.40 [2.04, 3.00]	2.30 [1.89, 3.10]	0.328
HDL-C (median [IQR])	1.01 [0.89, 1.13]	1.02 [0.91, 1.08]	0.930
D_D (median [IQR])	234.00 [124.00, 476.23]	372.00 [228.50, 848.50]	<0.001
PT (median [IQR])	12.17 [11.00, 14.00]	13.90 [12.60, 15.10]	<0.001
PTA (median [IQR])	99.15 [90.00, 108.00]	88.03 [79.00, 102.00]	0.001
INR (median [IQR])	1.00 [0.94, 1.07]	1.07 [0.98, 1.15]	<0.001
FIB (median [IQR])	3.31 [2.82, 3.95]	3.68 [3.06, 4.25]	0.031
APTT (median [IQR])	30.30 [28.33, 32.60]	30.60 [28.90, 33.50]	0.352
TT (median [IQR])	15.00 [13.80, 16.55]	14.61 [13.95, 16.20]	0.746
Duration (median [IQR])	525.00 [274.00, 929.50]	301.00 [88.50, 809.00]	0.004
Gende*r* = 2 (%)	115 (21.0)	14 (22.2)	0.871
Rutherford (%)			0.006
0	3 (0.5)	3 (4.8)	
1	24 (4.4)	3 (4.8)	
2	88 (16.1)	4 (6.3)	
3	185 (33.8)	15 (23.8)	
4	118 (21.6)	17 (27.0)	
5	129 (23.6)	21 (33.3)	
Smoking = 1 (%)	364 (66.5)	37 (58.7)	0.262
Diabetes = 1 (%)	243 (44.4)	32 (50.8)	0.352
Hypertension = 1 (%)	325 (59.4)	43 (68.3)	0.221
Heart_disease = 1 (%)	110 (20.1)	20 (31.7)	0.050
Heart_failure = 1 (%)	10 (1.8)	2 (3.2)	0.357
Brain_diseases = 1 (%)	122 (22.3)	18 (28.6)	0.270
Chronic_kidney_disease_staging = 1 (%)	12 (2.2)	2 (3.2)	0.647
Anemia = 1 (%)	11 (2.0)	3 (4.8)	0.168
Hyperhomocysteinemia = 1 (%)	29 (5.3)	4 (6.3)	0.766
Hyperlipidemia = 1 (%)	28 (5.1)	1 (1.6)	0.347
disease_category (%)			0.292
Endovascular intervention	295 (53.9)	28 (44.4)	
Open surgery	179 (32.7)	25 (39.7)	
Hybrid surgery	52 (9.5)	9 (14.3)	
Unknown type	21 (3.8)	1 (1.6)	
Anatomical lesion distribution, *n* (%)			0.086
Aortoiliac lesions	178 (32.5)	24 (38.1)	
Femoropopliteal lesions	421 (77.0)	59 (93.7)	
Below-the-knee lesions	226 (41.3)	33 (52.4)	
Other arterial lesions	6 (1.1)	1 (1.6)	
Intervention indication, *n* (%)			0.168
Intermittent claudication	198 (36.2)	21 (33.3)	
Chronic limb-threatening ischemia (CLTI)	349 (63.8)	42 (66.7)	

Age (years); BMI (kg/m^2^); LVEF (%);WBC (×10^9^/L); RBC (×10^12^/L); HGB (g/L); HCT (%); MCV (fL); MCH (pg); MCHC (g/L); RDW-CV (%); RDW-SD (fL); PLT (×10^9^/L); MPV (fL); PDW(fL); ESO/BASO/NEUT/LYMPH/MONO (×10^9^/L); TP/ALB/GLB (g/L); ALT/AST (U/L); Cr (*μ*mol/L); FBG/Urea/Ca/TC/TG/LDL-C/HDL-C (mmol/L); D-D (ng/mL); PT/APTT/TT (s); PTA (%); INR (); FIB (g/L); Duration (min). Categorical variables: Gender (1 = male, 2 = female); Rutherford (0–5 grades for limb ischemia);Smoking/Diabetes/Hypertension/Heart_disease/Heart_failure/Brain_diseases (1 = yes, 0 = no); Chronic_kidney_disease_staging (1–5 grades); Anemia (1 = HGB<120 g/L male/<110 g/L female, 0 = n o); Hyperhomocysteinemia (1 = homocysteine>15 μmol/L, 0 = no); Hyperlipidemia (1 = yes, 0 = no); All laboratory indices: preoperative fasting venous blood tests.

#### 2.1.1 Missing data handling

Variables with a missing rate >30% were excluded. Missing values <30% were imputed using the random forest method (missForest package). This study was adopted 5-fold cross-validation for imputation optimization, with the maximum iteration number set to 100 and the termination threshold of relative error <0.001 (see Supplementary Table S1 for missing value statistics).

#### 2.1.2 Outlier handling

Extreme values were identified and adjusted using Tukey's fences (1.5 × interquartile range, IQR).

#### 2.1.3 Feature encoding

Categorical variables were one-hot encoded; continuous variables were standardized via Z-score normalization.

### 2.2 Traditional statistical analysis

Statistical analyses were performed using SPSS 27.0.1 and R 3.6.0. The Shapiro–Wilk test was used to assess normality of continuous variables. Normally distributed data were reported as mean ± standard deviation, and non-normally distributed data as median (interquartile range). Categorical variables were presented as counts and percentages. Group comparisons were conducted using Student's *t*-test, Mann–Whitney *U*-test, or chi-square test, as appropriate.

Initially, the association between NLR and mortality was evaluated using Kaplan–Meier analysis, accompanied by the Log-rank test. Prior to this analysis, NLR values were dichotomized based on the median. Subsequently, univariate and multivariate Cox proportional hazards regression models were constructed to analyze the relationships between NLR, other variables, and all-cause mortality. The multivariate Cox proportional hazards model included covariates selected based on clinical relevance and univariate results, comprising Rutherford classification, age, albumin (ALB), hemoglobin (HGB), history of heart disease (Heart_disease), and NLR. Results are presented as hazard ratios with 95% confidence intervals.

### 2.3 Machine learning model development

#### 2.3.1 Data preprocessing and partitioning

The dataset was randomly divided into a training set (*n* = 427) and an independent test set (*n* = 183) in a 7:3 ratio, ensuring balanced proportions of mortality cases between the two groups. All continuous features were standardized (Z-score normalization).

#### 2.3.2 Model training and comparison

In this study, six machine learning models, including logistic regression, decision tree, support vector machine (SVM), random forest, Adaboost, and XGBoost, were constructed and trained on the training set using 5-fold cross-validation. During the model training process, hyperparameters for each model were optimized through Bayesian optimization combined with 5-fold cross-validated grid search to enhance model generalization performance.

#### 2.3.3 Model performance evaluation

The assessment of model performance was conducted across multiple dimensions. Discriminative ability was evaluated using the area under the receiver operating characteristic curve(AUC) along with its 95% confidence interval, and statistically significant differences in AUC values between different models and traditional clinical stratification tools were compared using DeLong's test. All statistical tests in this study were two-tailed with a unified significance level of *α* = 0.05, and a *P* value less than 0.05 was considered statistically significant. Calibration was comprehensively assessed using the Brier score and calibration curves. Classification performance was determined by calculating precision, recall, F1 score, and specificity at the optimal Youden index threshold. Clinical utility was evaluated using decision curve analysis (DCA).

#### 2.3.4 Model interpretation

To investigate the contribution of each individual feature to mortality prediction in PAD patients, we computed SHAP values from the random forest model using Tree SHAP. The SHAP values, representing the individual contributions to the log-odds of the prediction, were visualized using the R package shapviz (v.0.9.6). SHAP facilitates both local and global interpretability. We leveraged SHAP to provide an explanation for our predictive model, which incorporated relevant risk factors associated with mortality in PAD patients. To identify the primary predictors of all-cause mortality within the patient cohort, we calculated the importance of feature rankings in the final model.

All statistical analyses and machine learning modeling in this study were performed using standard built-in algorithms in SPSS 27.0.1 and R 3.6.0 software. The software packages utilized included: survival/survminer (for cox regression and kaplan-meier analysis), caret/ranger/XGBoost/e1071/adabag/rpart (for training machine learning models), shapr (for SHAP analysis), missForest (for multiple imputation), and pROC (for ROC curve analysis). All statistical tests adopted a two-tailed design with a significance threshold set at *α* = 0.05.

This study was approved by the Ethics Committee of the Second Hospital of Shanxi Medical University. Given its retrospective design and use of anonymized routine clinical data, informed consent was waived. All procedures were conducted in accordance with the Declaration of Helsinki.

## 3 Results

### 3.1 Baseline characteristics

The study included 610 patients, of whom 63 died during the follow-up period. The cause-specific mortality was summarized as follows: 43 patients died of cardiovascular and cerebrovascular diseases (68.3%), 17 patients died of severe infection/sepsis (27.0%), and the remaining 3 patients died of other causes (4.8%). Baseline characteristics of survivors and non-survivors are compared in Table 1. Compared to survivors, non-survivors were significantly older (74.49 ± 8.72 vs. 67.38 ± 10.09 years, *P* < 0.001), had higher levels of WBC (7.92 vs. 6.97, *P* < 0.01), neutrophils (5.40 vs. 4.40, *P* < 0.01), NLR (3.6 vs. 2.40, *P* < 0.001), creatinine (78.55 vs. 71.00, *P* < 0.05), and urea (6.70 vs. 6.00, *P* < 0.05), suggestive of worse renal function, and had lower levels of albumin (36.00 vs. 38.70, *P* < 0.001). Non-survivors had a higher proportion of patients classified as Rutherford grade 4 (27.0% vs. 21.6%) and grade 5 (33.3% vs. 23.6%). In contrast, body mass Index (BMI), left ventricular ejection fraction (LVEF), platelet count (PLT), mean platelet volume (MPV), platelet distribution width (PDW), eosinophilic granulocyte (ESO), basophil granulocyte (BASO), monocyte (MONO), total protein (TP), alanine aminotransferase (ALT), aspartate aminotransferase (AST), triglycerides (TG), low-density lipoprotein cholesterol (LDL-C), high-density lipoprotein cholesterol (HDL-C), activated partial thromboplastin time (APTT), thrombin time (TT), and fasting blood glucose (FGB) were balanced between the groups, with no significant differences observed (*P* > 0.05).

### 3.2 Traditional statistical analysis

**Figure 1:**
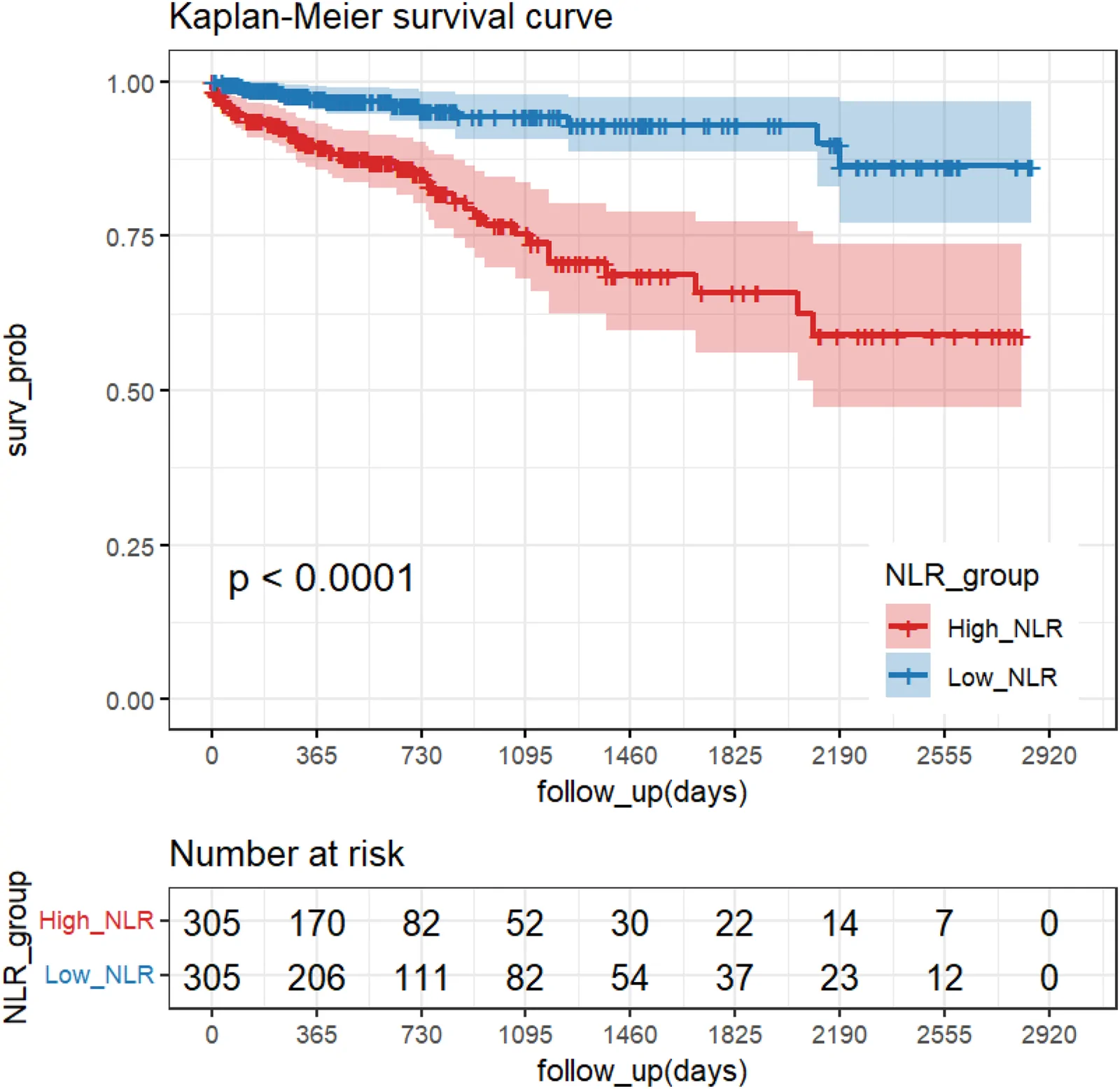
Kaplan–meier survival curve (grouped by NLR: Low-Medium vs. High).

Patients were divided into low-NLR and high-NLR groups based on the median NLR of 2.51 for survival analysis. Kaplan–Meier curves (Figure 1) showed that the low-NLR group maintained a continuously higher survival rate throughout follow-up, with a gradually widening survival gap between the two groups; the Log-rank test confirmed a significant survival difference (*P* < 0.0001). Univariate analysis verified that elevated NLR was closely associated with poor prognosis.

**Figure 2:**
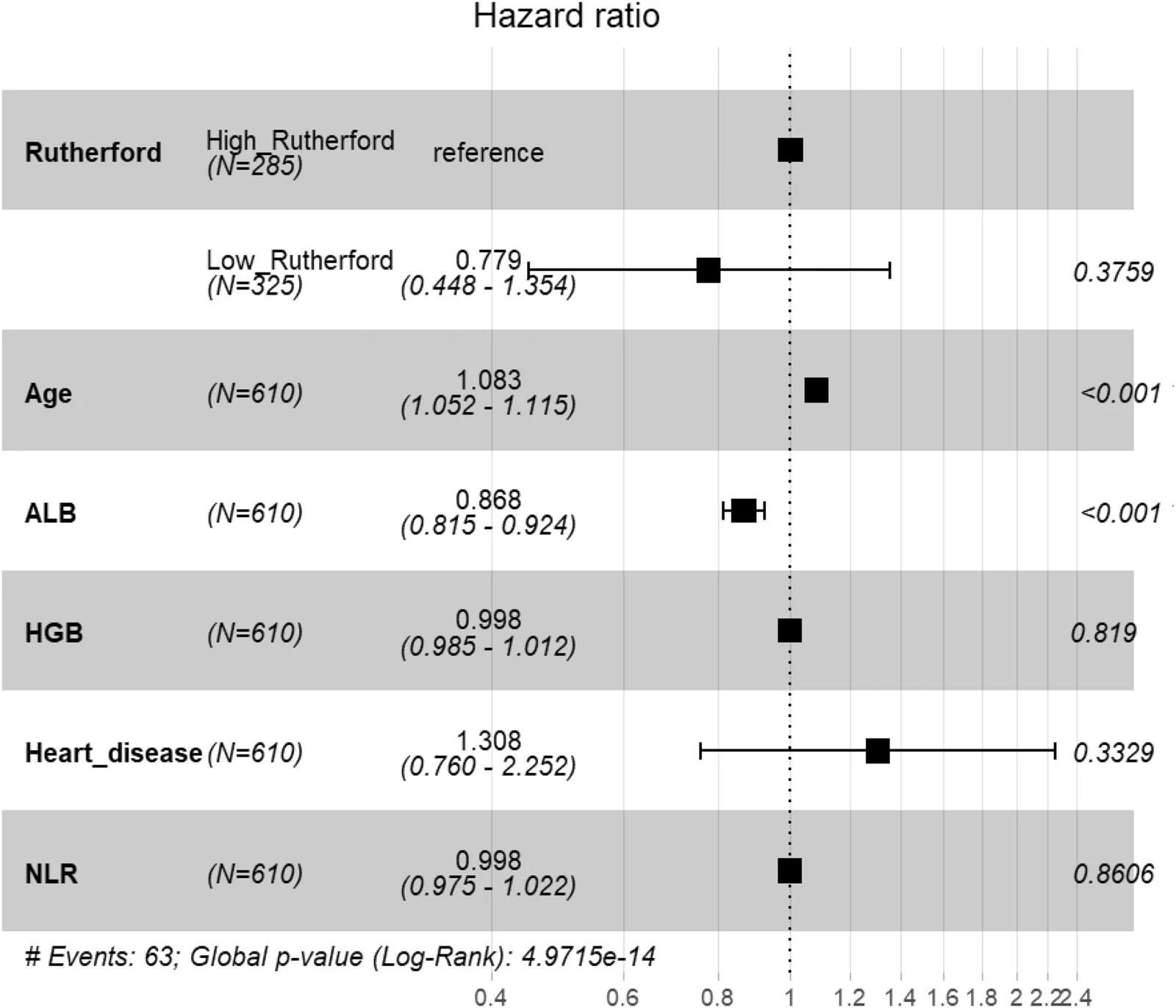
Multivariate Cox regression forest plot.

To identify independent mortality predictors, we established a multivariate Cox regression model (Figure 2) incorporating NLR, Rutherford classification, age, ALB, hemoglobin and history of heart disease. The overall model was statistically significant (*P* < 0.0001). The results demonstrated that age (HR = 1.083, 95%CI: 1.052–1.115, *P* < 0.001) and ALB (HR = 0.868, 95%CI: 0.815–0.924, *P* < 0.001) were independent prognostic factors. Advanced age and decreased ALB correlated with higher mortality risk. After adjusting for confounders, Rutherford classification showed no significant predictive value for mortality (HR = 0.779, 95%CI: 0.448–1.354, *P* = 0.3759), which was consistent with its low AUC of 0.576 for mortality prediction. Likewise, hemoglobin, heart disease history and NLR failed to serve as independent linear predictors in the multivariate model. These results implied that the prognostic role of NLR was masked by stronger predictors such as age and ALB under linear statistical assumptions.

### 3.3 Machine learning model performance

Although traditional multivariate Cox regression indicated that NLR was not an independent predictor of mortality (*P* = 0.8606), considering the frequent presence of nonlinear or threshold effects between inflammatory markers and clinical outcomes, the linear assumptions of conventional models may have limited the full assessment of NLR's predictive value. To overcome this limitation, this study developed and evaluated the performance of six machine learning models.

#### 3.3.1 Model performance comparison

As shown in Table 2, random forest demonstrated the best discriminative performance, achieving an AUC of 0.814 (95% CI: 0.712–0.916), which was significantly higher than the other models (*P* < 0.05). Logistic regression and XGBoost (optimal threshold) ranked second and third with AUC values of 0.754 and 0.737, respectively. Notably, XGBoost exhibited the highest performance in sensitivity, reaching 0.750, indicating its ability to identify 75% of the actual mortality cases, which holds significant clinical importance for early screening and intervention.

**Table 2:** Performance comparison of different machine learning models.

Model	AUC	Sensitivity	Specificity	Precision	F1 Score
XGBoost (Optimal Threshold)	0.737	0.750	0.671	0.179	0.289
Random Forest	0.814	0.125	0.994	0.667	0.211
Logistic Regression	0.754	0.562	0.784	0.200	0.295
SVM	0.727	0.062	0.857	0.233	0.105
AdaBoost	0.710	0.188	0.902	0.238	0.250
Decision Tree	0.529	0.188	0.802	0.200	0.171

**Figure 3:**
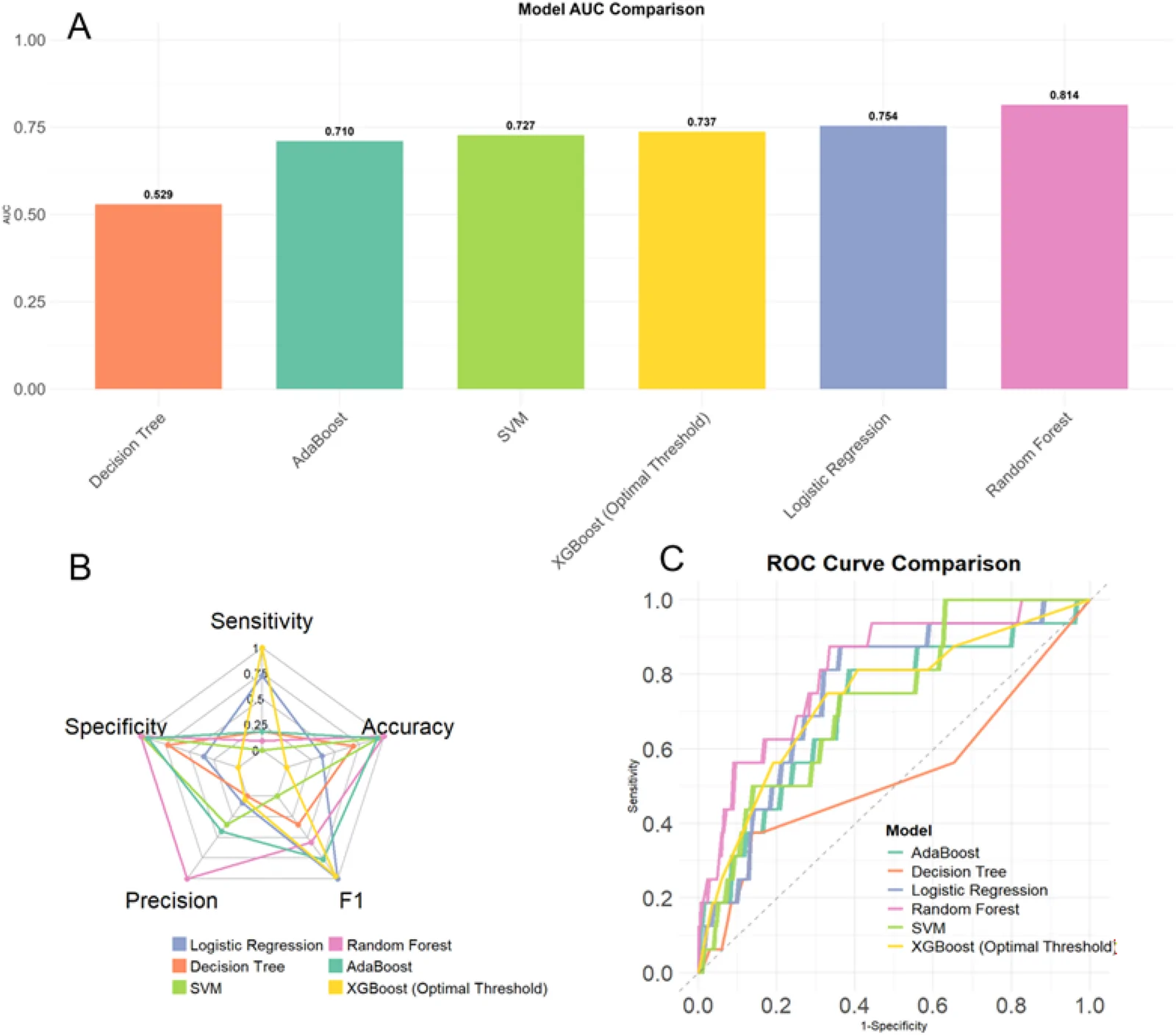
Visual performance analysis. **(A)** Model AUC comparison. **(B)** Model performance radar chart. **(C)** ROC curve comparison.

To visually compare the performance characteristics of each model, we conducted three types of visual analyses (Figure 3). The AUC bar chart comparison (Figure 3A) clearly illustrates the differences in AUC values among the models. The random forest bar is the highest, whereas the decision tree bar is significantly lower than the others, indicating its substantially inferior discriminative ability. The multi-indicator radar chart analysis (Figure 3B) provides a comprehensive assessment of model performance across five dimensions: sensitivity, specificity, precision, F1 score, and accuracy. Random forest exhibits a larger and more balanced polygon area, demonstrating its consistent performance across multiple metrics. In contrast, the SVM polygon shows a noticeable indentation in the sensitivity dimension, reflecting its lower capability in case identification. The ROC curve comparison (Figure 3C) illustrates the true positive rate and false positive rate variations of each model at different thresholds. The Random Forest ROC curve is closest to the upper-left corner, indicating its optimal discriminative ability in achieving a high true positive rate while maintaining a low false positive rate. Notably, XGBoost performs prominently in the high-sensitivity region (upper-left portion of the curve), which is of critical importance for avoiding missed diagnoses.

#### 3.3.2 Model interpretability and NLR contribution analysis

**Figure 4:**
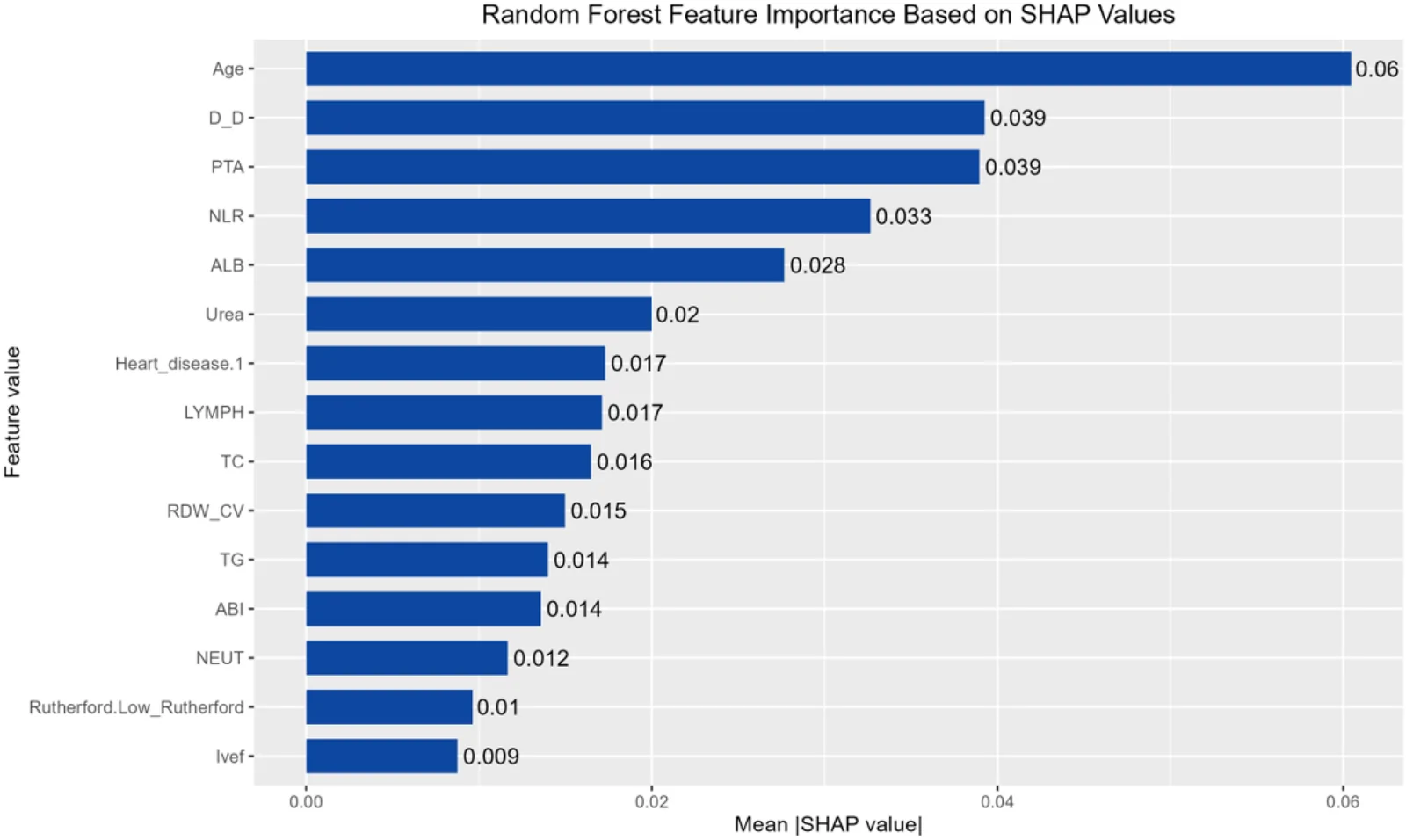
Random forest feature importance based on SHAP values.

Using SHAP, we delved into the contribution of each feature in the optimal model. As illustrated in Figure 4, age was the most important feature for predicting mortality risk (mean SHAP = 0.0404), followed by PTA (0.0131) and Rutherford classification (0.0093). NLR ranked 6th in importance within the Random Forest model (mean SHAP = 0.0015). Although its contribution value was relatively modest, it was notably higher than its performance in the traditional Cox regression model (*P* = 0.8606).

**Figure 5:**
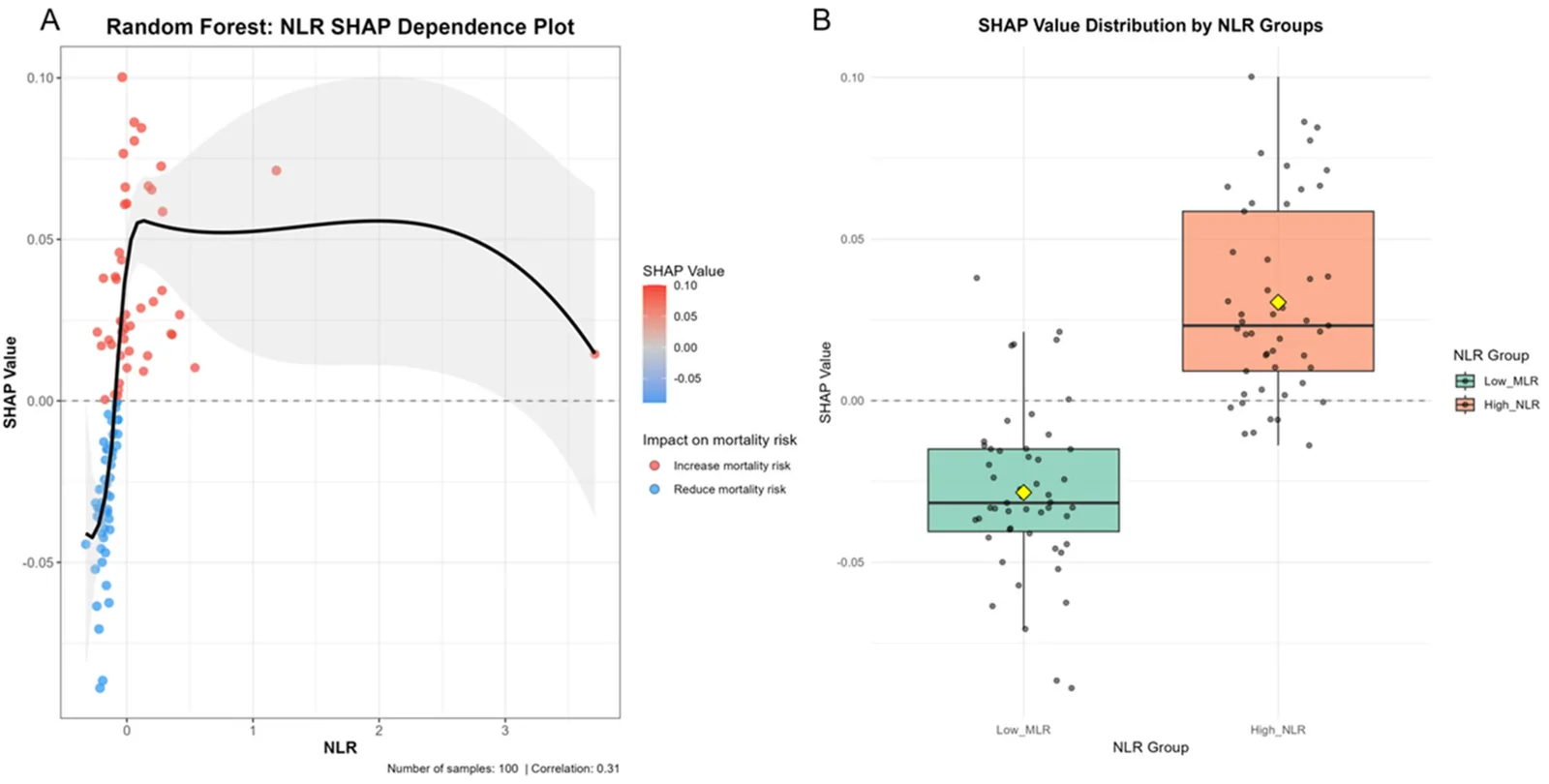
SHAP-based interpretation of NLR's contribution to mortality risk in random forest model. **(A)** Random forest: NLR SHAP dependence plot (shows the contribution of NLR values to the prediction of mortality risk). **(B)** SHAP value distribution by NLR groups (compares SHAP value distributions between NLR <2.51 and NLR ≥2.51).

The SHAP dependence plot (Figure 5A) revealed a distinct nonlinear threshold relationship between NLR and mortality risk. Within the low NLR range, rising NLR rapidly increased mortality risk; after exceeding a critical threshold, the risk contribution tended to plateau. This nonlinear characteristic cannot be captured by conventional linear models, which explains why NLR lost statistical significance in multivariate Cox analysis. Boxplots and scatter plots of SHAP values (Figure 5B) further validated that the high-NLR group exerted an overall stronger positive effect on mortality risk.

The SHAP interpretability analysis not only quantifies the predictive contribution of NLR in the Random Forest model, but also, more importantly, uncovers the complex nonlinear dependency between NLR and mortality risk. These findings explain the attenuation of NLR's prognostic significance from univariable Kaplan–Meier analysis to multivariable Cox modeling. This phenomenon likely stems from the inability of standard regression frameworks to accommodate the non-linear and complex interaction effects inherent in the underlying biology. Through explainable machine learning, this study successfully identifies and visualizes the hidden predictive value of NLR, providing a more nuanced perspective for understanding its role in the prognosis of peripheral artery disease.

#### 3.3.3 Decision curve analysis

**Figure 6:**
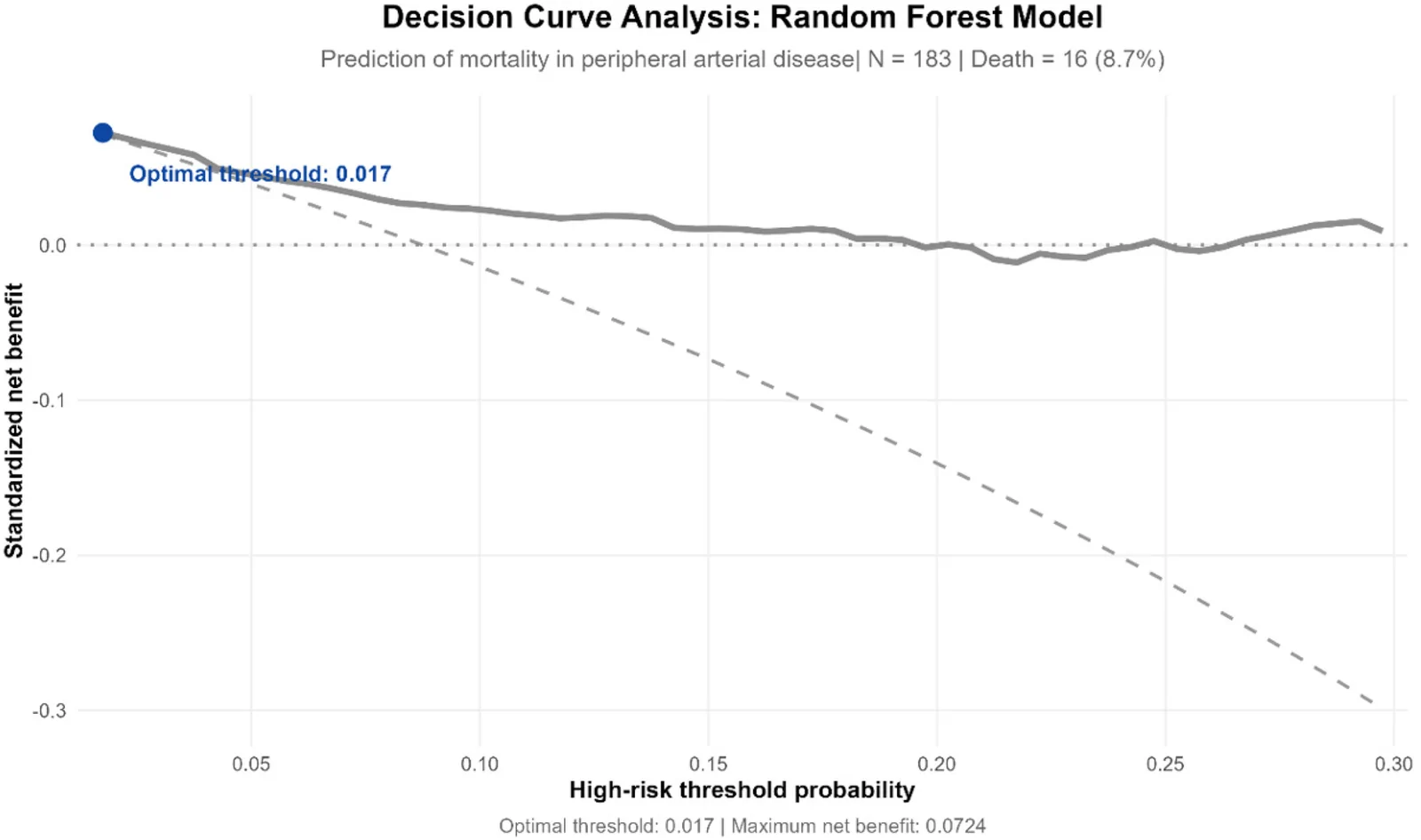
Decision curve analysis: random forest model.

We further evaluated the clinical net benefit of the Random Forest model across different risk thresholds using DCA. The DCA results are presented in Figure 6. Across the threshold probability range of 0.01 to 0.30, the decision curve for the Random Forest model consistently remained above the reference lines for “treat all” and “treat none”, indicating that applying this model to guide clinical decisions yields a positive standardized net benefit throughout this threshold range. The model achieved its maximum net benefit of 0.0724 at a threshold probability of 0.017. The DCA curve indicates that the optimal threshold for intervention is a predicted mortality probability of 1.7%. At this cutoff, adopting the model for clinical decision-making yields a substantial net benefit. Specifically, compared to the “treat all” and “treat none” strategies, this approach prevents approximately 7.2 unnecessary interventions per 100 patients, without compromising survival outcomes. Notably, this relatively low optimal threshold aligns with the lower actual mortality rate observed in the study cohort (8.7%), suggesting the model's practical value in identifying early-stage risk and avoiding missed diagnoses.

This random forest model achieved strong predictive accuracy (AUC: 0.814) and was rendered interpretable via SHAP analysis, which quantified the non-linear impact of variables such as age and NLR. Importantly, DCA confirmed that applying this model at reasonable decision thresholds can optimize clinical decision-making by maximizing net benefit for postoperative PAD management. This offers robust evidence for translating machine learning prediction models into practical tools to support clinical decision-making. Next research could further refine risk intervention thresholds applicable to different clinical scenarios, facilitating the model's clinical translation and application.

## 4 Discussion

PAD imposes a substantial global cardiovascular burden, characterized by high postoperative mortality rates that necessitate precise risk stratification (18–20). While inflammation is a known driver of atherosclerotic progression, the utility of inflammatory markers like NLR remains inconsistent in PAD prognostication, largely because traditional statistical models rely on linear assumptions that fail to capture complex biological interactions (21). To address this gap, this study constructed and validated six machine learning models to predict postoperative all-cause mortality in PAD patients and utilized the SHAP framework to elucidate the black-box mechanisms of the optimal algorithm.

This study successfully constructed and validated an interpretable random forest model for predicting postoperative all-cause mortality in PAD patients, achieving robust discriminative performance (AUC = 0.814) and demonstrating that the prognostic value of NLR follows a distinct threshold-dependent pattern rather than a linear relationship. A major strength of this study lies in its utilization of a substantial single-center cohort (*n* = 610) from a tertiary care hospital in China, which provides sufficient statistical power to explore complex nonlinear relationships and reduces sampling bias compared to numerous previous investigations limited by smaller sample sizes.

Consistent with previous literature, our study confirmed that advanced age and hypoalbuminemia are powerful independent predictors of long-term mortality in PAD patients. Aging represents an irreversible cumulative risk factor characterized by progressive vascular endothelial dysfunction, increased arterial stiffness, and weakened systemic stress tolerance, which substantially elevates postoperative cardiovascular and all-cause mortality risks (22, 23). Similarly, serum albumin reflects systemic nutritional status, inflammatory burden, and visceral protein reserve. Consistent with prior vascular surgery studies, reduced ALB indicates chronic malnutrition, persistent systemic inflammation, and impaired tissue repair capacity, all of which significantly increase postoperative mortality risk (24). Notably, although conventional clinical grading systems such as the Rutherford classification are widely used for evaluating PAD limb ischemia severity, our further quantitative analysis demonstrated that Rutherford classification exhibited only limited predictive efficacy for all-cause mortality (AUC = 0.576, 95% CI: 0.511–0.640) and failed to serve as an independent prognostic factor after multivariable adjustment (HR = 0.779, *P* = 0.3759). In contrast, our machine learning model integrates multidimensional systemic clinical variables and effectively captures nonlinear interactions, yielding superior prognostic performance and evident incremental value over traditional grading tools.

Of particular importance, this study identified a prominent nonlinear threshold-dependent prognostic effect of NLR, which provides novel mechanistic insights to resolve previous inconsistent conclusions regarding NLR in PAD prognosis. Most traditional clinical studies adopted linear regression or Cox models and assumed a monotonous linear association between NLR and mortality, leading to conflicting and inconsistent results across different cohorts (25, 26). The underlying pathophysiological mechanism of this nonlinear threshold effect can be explained by the dual biological roles of systemic inflammation in atherosclerotic disease progression. At low-to-moderate NLR levels, mild systemic inflammation may promote compensatory vascular repair, endothelial regeneration, and adaptive immune responses, without significantly increasing mortality risk (27). However, once NLR exceeds a critical threshold, persistent hyper-inflammation triggers a series of pathological cascades, including endothelial barrier destruction, accelerated atherosclerotic plaque instability, increased oxidative stress, systemic microcirculatory dysfunction, and immune homeostasis imbalance (14, 28). Excessively elevated neutrophil counts aggravate vascular inflammatory infiltration, while relatively insufficient lymphocyte counts reflect immune exhaustion and impaired anti-inflammatory protective mechanisms. This synergistic pathological change significantly increases the risks of postoperative cardiovascular events, multi-organ dysfunction, and long-term mortality. Therefore, the prognostic value of NLR is not linearly cumulative but manifests significant threshold saturation effects, which can only be precisely identified through nonlinear machine learning algorithms rather than conventional linear statistical methods.

Beyond clarifying the role of NLR, the SHAP framework identified age, PTA, and Rutherford classification as the top predictors of mortality, aligning with existing literature that emphasizes the cumulative risk of aging and the severity of limb ischemia. Notably, further quantitative comparison with the conventional Rutherford stratification system revealed that this traditional clinical grading tool exhibited only limited predictive efficacy for long-term all-cause mortality, with an AUC of 0.576 (95% CI: 0.511–0.640). After adjusting for multiple clinical confounding factors, high-grade Rutherford classification was not an independent prognostic factor for mortality (HR = 0.779, 95% CI: 0.448–1.354, *P* = 0.3759). These findings explicitly demonstrate that although Rutherford classification reflects the severity of limb ischemia, it cannot independently predict systemic mortality risk and shows poor discriminative ability in risk stratification. In contrast, the proposed machine learning model integrates multidimensional laboratory, clinical, and procedural variables and captures nonlinear variable interactions, thereby providing significantly superior predictive performance and incremental prognostic value over conventional empirical grading systems. Interestingly, while XGBoost showed slightly lower overall AUC, its superior sensitivity (0.750) highlights its potential for screening scenarios where avoiding missed diagnoses is paramount; conversely, the high specificity of random forest (0.994) makes it suitable for precise risk stratification to guide intensive interventions. DCA further corroborated the clinical utility of the Random Forest model, showing a positive net benefit across a threshold probability range of 0.01−0.30, which corresponds well with the low overall mortality rate (8.7%) in our cohort. This suggests that the model can guide clinicians to avoid approximately 7.2 unnecessary interventions per 100 patients compared to default strategies, without compromising survival outcomes.

From a translational clinical perspective, our interpretable random forest model possesses substantial practical application value for individualized preoperative risk evaluation and stratified clinical management of PAD patients undergoing revascularization. Different from traditional subjective empirical judgment and single-dimensional anatomical grading criteria, this model can generate objective and quantitative postoperative mortality risk scores based on routine preoperative indicators, enabling accurate preoperative risk stratification before surgery. In clinical practice, clinicians can input easily accessible preoperative clinical variables into the model to rapidly distinguish low-risk and high-risk patients, so as to formulate personalized perioperative management strategies and implement targeted early intervention.

For patients stratified as low-risk by the model, routine standardized perioperative management and regular postoperative follow-up are sufficient, which effectively avoids excessive medical intervention, reduces unnecessary medical resource consumption, and optimizes clinical resource allocation. For high-risk patients identified by the model, clinicians can conduct targeted preoperative optimization and intensified early intervention targeting core risk factors confirmed in this study. Specifically, individualized nutritional support can be actively implemented to correct hypoalbuminemia and improve systemic reserve function; targeted inflammatory regulation can be performed to improve excessive systemic inflammatory status and correct abnormal NLR threshold levels. Meanwhile, high-risk patients require strengthened perioperative hemodynamic monitoring, rigorous postoperative complication surveillance, and prolonged intensive follow-up, which facilitate early detection and timely management of potential cardiovascular events and organ dysfunction, and ultimately reduce long-term all-cause mortality.

Overall, this machine learning model converts experience-dependent clinical assessment into quantitative, precise, and interpretable risk evaluation. It compensates for the inherent defects of traditional PAD grading systems in systemic prognostic prediction, realizes refined preoperative risk stratification and hierarchical intervention, and provides reliable clinical decision-making evidence for individualized perioperative management of PAD patients, thereby helping improve patients' long-term prognostic outcomes.

Undeniably, several limitations warrant attention. As a single-center retrospective study, the generalizability of our model may be restricted, necessitating external validation in multicenter, prospective cohorts. In addition, this study only collected Rutherford classification data for conventional risk stratification comparison, while other mainstream PAD grading systems, including WIfI and GLASS classifications, were unavailable in our retrospective cohort. Additionally, while SHAP provides correlation, it does not establish causation; thus, the exact biological pathways linking specific NLR thresholds to mortality require further investigation through serial inflammatory profiling. Future research should focus on conducting multicenter prospective studies, integrating imaging data such as plaque characteristics, and developing dynamic web-based calculators to translate these ML insights into real-time clinical decision support.

Future research will be carried out in three main directions summarized as follows. First, the study team will perform multicenter prospective studies with enlarged sample sizes. WIfI and GLASS classifications will be included to further validate the generalizability of the model and compare its performance with mainstream clinical stratification systems. Second, additional data including imaging features and omics biomarkers will be integrated to optimize model performance. A visual clinical decision support system will also be developed, and relevant interventional trials will be conducted to confirm the clinical value of model-guided treatment. Third, detailed perioperative medication data, drug adjustment records and long-term medication adherence information will be collected in future prospective cohorts to reduce medication-related confounding and verify the robustness of our results.

## 5 Conclusions

This study utilized and validated an interpretable random forest model for predicting postoperative all-cause mortality in patients with PAD. Integrated SHAP analysis overcomes the black-box limitation of conventional machine learning and clarifies the nonlinear prognostic role of NLR. This model provides a robust, transparent, and clinically useful tool for individualized risk stratification, with the potential to improve postoperative management and outcomes in PAD.

## Data Availability

The datasets presented in this article are not readily available because the dataset contains sensitive patient clinical information and its access is restricted by the Ethics Committee of the Second Hospital of Shanxi Medical University to protect patient privacy. Requests to access the datasets should be directed to Lishuang Guo (guolishuang@sxmu.edu.cn) and Sheng Yan (578115246@qq.com).
