# Supplementary material for: Prediction of mortality risk in patients with peripheral artery disease using interpretable machine learning
Source: Front Cardiovasc Med. 2026 Aug 10;13:1879240. doi: 10.3389/fcvm.2026.1879240 (PMC13500288; doi:10.3389/fcvm.2026.1879240)
Supplement: Supplementary file 1 [file Table1.docx]

# Supplementary Materials

**Table S1 Missing Values Statistics Table**

| **Variable** | **Missing number** | **Missing rate (%)** |
| --- | --- | --- |
| **TNT** | 609 | 99.84 |
| **SLDL** | 567 | 92.95 |
| **APO_B** | 551 | 90.33 |
| **RBP** | 550 | 90.16 |
| **NT_proBNP** | 548 | 89.84 |
| **APO_A1** | 547 | 89.67 |
| **Mg** | 538 | 88.20 |
| **PA** | 537 | 88.03 |
| **fu_L_ABI** | 537 | 88.03 |
| **fu_R_ABI** | 537 | 88.03 |
| **P** | 520 | 85.25 |
| **CK** | 496 | 81.31 |
| **HBDH** | 495 | 81.15 |
| **LDH** | 492 | 80.66 |
| **UA** | 467 | 76.56 |
| **BNP** | 403 | 66.07 |
| **GGT** | 375 | 61.48 |
| **ALP** | 374 | 61.31 |
| **Intraoperative_bleeding** | 372 | 60.98 |
| **cTNI** | 363 | 59.51 |
| **LP(a)** | 351 | 57.54 |
| **MYO** | 343 | 56.23 |
| **HCY** | 334 | 54.75 |
| **CK_MB** | 317 | 51.97 |
| **CRP** | 284 | 46.56 |
| **TC** | 195 | 31.97 |
| **LDL_C** | 194 | 31.80 |
| **HDL_C** | 194 | 31.80 |
| **TG** | 193 | 31.64 |
| **ABI_left** | 191 | 31.31 |
| **ABI_right** | 190 | 31.15 |
| **LVEF** | 181 | 29.67 |
| **FBG** | 116 | 19.02 |
| **TT** | 53 | 8.69 |
| **Urea** | 52 | 8.52 |
| **GLB** | 45 | 7.38 |
| **Ca** | 45 | 7.38 |
| **Cr** | 43 | 7.05 |
| **TP** | 42 | 6.89 |
| **ALB** | 42 | 6.89 |
| **Target_vessel_status** | 39 | 6.39 |
| **Target_limb_status** | 39 | 6.39 |
| **Rutherford** | 35 | 5.74 |
| **ALT** | 33 | 5.41 |
| **AST** | 29 | 4.75 |
| **time** | 28 | 4.59 |
| **D-D** | 23 | 3.77 |
| **RDW_CV** | 20 | 3.28 |
| **NLR** | 19 | 3.11 |
| **RDW_SD** | 18 | 2.95 |
| **MPV** | 18 | 2.95 |
| **PDW** | 18 | 2.95 |
| **NEUT** | 18 | 2.95 |
| **LYMPH** | 18 | 2.95 |
| **PLT** | 17 | 2.79 |
| **MONO** | 17 | 2.79 |
| **PT** | 15 | 2.46 |
| **PTA** | 15 | 2.46 |
| **INR** | 15 | 2.46 |
| **RBC** | 14 | 2.30 |
| **WBC** | 13 | 2.13 |
| **HGB** | 13 | 2.13 |
| **HCT** | 13 | 2.13 |
| **MCV** | 13 | 2.13 |
| **MCH** | 13 | 2.13 |
| **MCHC** | 13 | 2.13 |
| **ESO** | 13 | 2.13 |
| **BASO** | 13 | 2.13 |
| **FIB** | 13 | 2.13 |
| **APTT** | 13 | 2.13 |
| **Rutherford_fu** | 6 | 0.98 |
| **BMI** | 5 | 0.82 |

*Notes:* ABI, ankle-brachial index; ALT, alanine aminotransferase; ALP, alkaline phosphatase; ALB, albumin; APO_A1, apolipoprotein A1; APO_B, apolipoprotein B; APTT, activated partial thromboplastin time; AST, aspartate aminotransferase; BASO, basophil count; BNP, brain natriuretic peptide; BMI, body mass index; Ca, serum calcium; CK, creatine kinase; CK_MB, creatine kinase-MB; cTNI, cardiac troponin I; Cr, creatinine; D-D, D-dimer; ESO, eosinophil count; FBG, fasting blood glucose; FIB, fibrinogen; fu, follow-up; GGT, gamma-glutamyl transferase; HCT, hematocrit; HBDH, α-hydroxybutyrate dehydrogenase; HCY, homocysteine; HGB, hemoglobin; HDL-C, high-density lipoprotein cholesterol; INR, international normalized ratio; LDL-C, low-density lipoprotein cholesterol; LDH, lactate dehydrogenase; LP(a), lipoprotein(a); LVEF, left ventricular ejection fraction; LYMPH, lymphocyte count; MCV, mean corpuscular volume; MCH, mean corpuscular hemoglobin; MCHC, mean corpuscular hemoglobin concentration; Mg, serum magnesium; MONO, monocyte count; MPV, mean platelet volume; MYO, myoglobin; NEUT, neutrophil count; NLR, neutrophil-to-lymphocyte ratio; NT_proBNP, N-terminal pro-brain natriuretic peptide; P, serum phosphorus; PA, prealbumin; PDW, platelet distribution width; PLT, platelet count; PT, prothrombin time; PTA, prothrombin time activity; RBP, retinol-binding protein; RBC, red blood cell count; RDW-CV, red blood cell distribution width-coefficient of variation; RDW-SD, red blood cell distribution width-standard deviation; SLDL, small dense low-density lipoprotein; TC, total cholesterol; TG, triglyceride; TP, total protein; TT, thrombin time; TNT, troponin T; UA, serum uric acid; GLB, globulin; WBC, white blood cell count. Rutherford_fu, Rutherford at follow-up; fu_L_ABI, Left ABI at follow-up; fu_R_ABI, Right ABI at follow-up.
